# Effects of Crime Type and Location on Park Use Behavior

**DOI:** 10.5888/pcd17.190434

**Published:** 2020-07-30

**Authors:** Oriol Marquet, S. Scott Ogletree, J. Aaron Hipp, Luis J. Suau, Candice B. Horvath, Alexander Sinykin, Myron F. Floyd

**Affiliations:** 1 Universitat Autònoma de Barcelona, Barcelona, Spain; 2 North Carolina State University, Raleigh, North Carolina; 3 Greensboro Police Department, Greensboro, North Carolina

## Abstract

**Introduction:**

Crime and the fear of crime can be a barrier to park use, and locations of crimes can have varied effects. Unsafe areas in or around the park, around the residence, or along the route to the park can alter park use behavior. Our study aimed to examine associations between objective measures of types and location of crimes and park use behaviors.

**Methods:**

In 2013 we surveyed a sample (N = 230) of residents in Greensboro, North Carolina, about park use, with responses matched to objective crime and spatial measures. We measured all crimes and violent crimes near home, near the closest park, and along the shortest route between home and park. By using ordered and binary logistic modeling, we examined the relationships between the locations of crime and park use and duration of park visit, park rating, and never visiting parks. Additional models included distance to the closest park.

**Results:**

Increased crime in parks and near home was associated with fewer park visits. Greater violent crime in all locations was related to fewer park visits. Park ratings were lower for parks with high violent crime rates.

**Conclusion:**

Given the importance of parks as settings for outdoor recreation and physical activity, crime may have a detrimental effect on physical activity and, therefore, public health.

**Summary**

**What is already known about this topic?**

Crime and the fear of crime can be a barrier to park use by adults, hindering the benefits parks can have for physical activity and health.

**What is added by this report?**

Using crime incident data, we examined the role crime locations (near home, near parks, and along routes to parks) play in park use.

**What are the implications for public health practice?**

Higher levels of crime near home and parks limit park use and residents’ rating of parks. Sources of crime must be considered if parks are to be a means of promoting physical activity in adults. Real and perceived crime can be a barrier to park use.

## Introduction

Fear of crime is a barrier to outdoor physical activity and park use among US adults (1,2). Given the importance of urban parks as settings for outdoor recreation and physical activity (3), the presence of crime may negatively affect the public’s health. Research has shown that violence is usually spatially clustered and tends to remain stable over time (4). Living in areas exposed to crime and violence is associated with trauma and mental health issues and is linked to psychological symptoms such as depression and anxiety (5,6). In addition, the presence of crime and violence may deter people’s use of parks, reducing the likelihood of their engaging in healthy behaviors.

Strong evidence exists that high-quality green spaces in residential environments are important for public health promotion (7), because both availability and quality of green spaces near the residence have positive and significant associations with park use and physical activity (8). However, having a park available does not necessarily increase park use (9), because this relationship may be affected by the presence of crime and perceptions of safety. Fear of crime may restrict physical activities and lead people to avoid places or situations they perceive to be unsafe. Crime can affect park use through 3 different pathways. First, living in areas with high crime may pose a barrier to engaging in outdoor activity and, instead, favor safe, interior environments. Second, researchers have explored how the presence of crime can hinder walking behavior, with the potential to reduce access to neighborhood parks (10). If walking is deemed dangerous, then access to a park would require driving or public transit, creating other types of constraints such as car availability and the availability of parking both at the beginning and end of the trip. Third, crimes that occur inside or near the park deter people from engaging in normal park activities. Parks can be gathering areas for gang members (11), and some park designs — with obstructed view-lines and places to hide — facilitate crime (12).

Evidence regarding crime and park use is limited; however, research in North Carolina found that parks located in neighborhoods with high crime indexes had less park use by all subpopulations except adolescents (13). Shinew et al found that adolescents avoided gang territories and other high-crime areas for organized sports and outdoor recreation (11). Han et al found that violent crimes had a stronger negative effect on park use than property crimes and observed that adults were more likely to change their park visiting habits than younger people (14). Marquet et al used crime reports to associate crime changes and park attendance in 3 different temporal thresholds (more than 1 week, 1 month, or 3 months), and found that violent crime affected park use and the number of children that used parks (15).

Most studies investigating crime and park use measured self-assessed perceptions regarding safety (10). Using objective measures of crime prevents self-reporting bias (8). Understanding how safety affects park use can facilitate translation of research findings into interventions (16). Previous studies have been limited by the use of aggregate measures of safety perception or crime indexes, which may mask true associations between crime and park use (17). Using disaggregated measures of crime that account for different levels of violence can help to overcome some of these limitations.

Similarly, most studies use measures of crime indexes taken at an aggregated level and do not differentiate by locations of the crimes. Just as the effects of different domains of safety and types of crime vary, different locations of crimes can have different effects (11,18). Although aggregated crime around the residence might be the main factor setting the perception of insecurity, the presence of unsafe areas around the park or along the route to the park can also alter park use. Our study aimed to examine associations between objective measures of types and location of crimes and park use behaviors.

## Methods

Our study was part of a data collection effort undertaken in 2013 in Greensboro, North Carolina. Greensboro, the third largest city in North Carolina, has a population of 269,666 (19) and covers an area of 127 square miles. In 2013 the median household income in Greensboro was $41,050 (20), and the racial/ethnic composition of the population was 47.8% white, 41.6% African American, 7.5% Hispanic of any race, 4.7% Asian, and 5.9% other or more than 2 races (19). The city was highly segregated and had a large proportion of minority neighborhoods (21). The park system dates to 1933 and comprises 170 properties (parks, gardens, and special facilities).

### Study sample

We conducted a mail survey to obtain data on park use and on rating of parks, physical activity, self-rated health, height and weight, and demographics from a sample of adults randomly drawn from residential addresses located within a quarter mile of one of 21 Greensboro parks. Parks were purposely selected to capture both low-income neighborhoods (n = 7) and middle-to-high income neighborhoods (n = 14) with only 1 park within a quarter-mile radius. The selected parks were described by the city as neighborhood parks from 5 to 15 acres in size that served residences in a half-mile radius (a 10-minute walk) (22). Residential addresses in the study areas were obtained from county GIS parcel data provided by the local government. We generated 893 residential addresses from street addresses rather than from owners’ addresses to target people who either rent or own a home in a quarter-mile of each park. Areas surrounding parks in low-income areas were oversampled by 10% to better capture low-income residents. The protocol for the mail survey followed a modified version of Dillman’s Tailored Design Method (23). We sent a letter to 893 residences explaining the study and including a questionnaire and a postage-paid return envelope. A reminder post card was sent to nonrespondents approximately 2 weeks after the first mailing. Approximately 2 weeks after that reminder post card, we mailed a second letter, questionnaire, and postage-paid return envelope to nonrespondents. As an incentive, respondents had a chance to win one of four $50 gift cards in a raffle. Of the 893 questionnaires mailed, 145 were returned as undeliverable. Of the 748 deliverable surveys, 235 were completed and returned and 5 were eliminated as incomplete or as duplicate questionnaires from the same address. The final count of 230 usable questionnaires yielded a response rate of 31%. Early respondents were compared with late respondents to investigate nonresponse bias. No significant difference in demographic characteristics was found between these 2 groups. By using the respondent addresses obtained from county parcel GIS files and a survey question asking the name of the park closest to the home, we were able to add contextual census-based information at the block group level regarding average population density and median household income for each participating household. We used ArcGIS v10.3.1 (Esri) for all spatial computations.

### Measures

**Park use.** Four survey questions measured park use: frequency of park visits (How often do you usually visit the park closest to your home?), duration of park visits (On a typical day when you visit the park closest to your home, how long do you usually stay?), and park rating (Overall how would you rate the parks in your neighborhood?). Responses were all ordered variables with no multiple responses allowed. We also included a bivariate item that asked whether respondents never went to their closest parks at all.

**Crime.** All offenses classified under the Summary Reporting System of the Federal Bureau of Investigation’s Criminal Justice Information Service and the Uniform Crime Reporting System are classified either as Part I or Part II offenses (24). Part I offenses are criminal homicide, rape, robbery, aggravated assault, burglary, larceny, motor vehicle theft, arson, and human trafficking; all crimes are classified as Part II offenses. We used Greensboro Police Department public data on all offenses reported in the city in 2012, provided as geolocated incident points. A total of 31,845 offenses were reported by the department, of which 14,789 (46.4%) were classified as Part 1 offenses. We used ArcGIS to count the number of all offenses and Part I offenses only that occurred within a 0.5 mile (10-minute walk) buffer of each participant residence (street network buffer) (25,26). Because the police report all crimes and offenses that occur within parks at the nearest address, we created a buffer of 15 meters around each park boundary and counted how many offenses and Part I-only offenses occurred immediately around or within the park boundaries. Finally, we used the ArcGIS network analyst function to calculate the shortest routes from each respondent’s residence to their closest-to-home park, assuming this to be the walking route from home to park. All offenses and Part I-only offenses that fell along this route were then counted. Overall we used 3 measures of crime location (around home, around park, and along the route from home to park) and 2 crime classifications (all crimes and Part I crimes only) as independent variables and to examine associations between crime type, crime locations, and park use behavior.

**Distance to park.** Distance to the closest park was calculated by using ArcGIS network analyst functions. We performed a sensitivity analysis correlating park use frequency with distance from home to the boundary of the park, distance from home to the centroid of the park, and distance from home to the closest park feature (such as playground or sports court). Correlation was strongest for distance to the park boundary (r[230] = −0.153, *P* = .02); therefore, we used distance to the park boundary as the main measure of distance.

### Statistical analysis

We used ordered logistic and binary logistic models to assess how type and location of crimes were associated with self-reported measures of park use frequency, time spent in the park, park rating, and never going to the park. We entered presence of crimes as a continuous dependent variable. To account for differences in neighborhood characteristics, all models were adjusted for the respondent’s age, sex, and race/ethnicity; the presence or absence of children in the household; and median household income (calculated on the basis of census data). Separate models were estimated for each type of crime (all crimes and Part I-only crimes) and for each location’s criteria (crimes around the home, crimes around the park, crimes along the shortest route from home to the park). We then used distance to the park as an additional independent variable. For the distance to park variable we built 3 separate models: a baseline model without adjusting for crime, a second model adjusting for all crimes, and a third model adjusting for Part I crimes only. All crime variables and distance variables were standardized. We used odds ratios instead of coefficients for clarity in interpretation.

Because the measures of frequency of park use, duration of park use, and park rating were ordinal, we used an ordered logistic regression to model them, whereas we used a binary logistic model for never going to the park. We used Stata SE v14.2 (StataCorp) for all statistical analyses.

## Results

Our sample (N = 230) was predominantly women (73.9%) and consisted of older adults (mean age, 54.5 y; standard deviation [SD], 16.4); 57.8% identified themselves as African American, and 68.3% reported children in the home (Table 1). Most participants had at least some college, a college degree, or a professional degree (78.9%) and reported their health as very good or excellent (60.8%). Participants lived in areas slightly more densely populated than the Greensboro average (4,030 people/mi^2^ vs 2,131 people/mi^2^) (Table 2) and in areas with higher median household income than the overall Greensboro median ($61,859 vs $40,361). On average, participants had a park within a quarter mile of their home (mean, 0.28 mi; SD, 0.16 mi). Participants also lived in areas with more crime than the city average (173 crimes per 1,000 population vs 49.28 crimes per 1,000 population for the city). The areas around parks had slightly higher crime rates than the residential neighborhoods of respondents, both in terms of all crimes and in terms of Part I crimes. Despite these numbers, 43.9% of our sample perceived parks in their neighborhood to be very good or excellent (Table 1).

**Table 1:** Characteristics, Survey of Respondents (N = 230)^a^ on Relationship Between Park Use and Crime, Greensboro, North Carolina, 2013

Characteristic	No. (%)
**Demographics**	
**Sex, n = 218**	
Female	161 (73.9)
Male	57 (26.1)
**Age, y, n = 217**	
18–34	27 (12.4)
35–49	53 (24.4)
50–64	78 (35.9)
≥65	59 (27.2)
**Race/ethnicity, n = 217**	
Non-Hispanic white	83 (38.1)
African American	126 (57.8)
Other	9 (4.1)
**Children in household, n = 218**	
Yes	149 (68.3)
No	69 (31.7)
**Education, n = 218**	
High school diploma or less	46 (21.1)
Some college or associate's degree	69 (31.7)
Bachelor degree/professional license	103 (47.2)
**Self-reported general health, n = 222**	
Poor or fair	30 (13.5)
Good	57 (25.7)
Very good or excellent	135 (60.8)
**Body mass index (weight in kg/height in m^2^), n = 213**	
Normal (18.5 to <25)	74 (34.7)
Overweight (25 to <30)	76 (35.7)
Obese (≥30)	63 (29.6)
**Park Behavior**	
**Visit frequency, n = 229**	
Never	27 (11.8)
Rarely	69 (30.1)
Couple times a month	41 (17.7)
Once a month	31 (13.5)
Few times a week	47 (20.5)
Daily	14 (6.1)
**Length of stay, n = 185**	
<15 min	29 (15.7)
15–30 min	70 (37.8)
31–60 min	49 (26.5)
>1h–<2 h	26 (14.1)
2–3 h	11 (5.9)
**Park rating, n = 228**	
Poor	22 (9.6)
Fair	47 (20.6)
Good	59 (25.9)
Very good	74 (32.5)
Excellent	26 (11.4)

a Number of respondents in each category varies by the number of valid responses to the question.

**Table 2:** Spatial Characteristics of Sample Households and Neighborhoods, Survey of Respondents (N = 230) on Relationship Between Park Use and Crime, Greensboro, North Carolina, 2013^a^

Variable	Neighborhood Around Residence	Neighborhood Around Park
Density (no. people/mi^2^)	4,030.7 (3,698.0)	4,358.3 (4,337.9)
Median household income, $	61,858.6 (32,464.5)	64,770.2 (33,443.6)
All crimes/1,000 people within block group^b^	173.0 (185.8)	191.1 (206.1)
Part 1 crimes^c^/1,000 people within block group^b^	89.7 (128.0)	99.1 (150.5)
Count of all crimes within 0.5 mile buffer^d^	322.7 (266.1)	550.2 (407.8)
Count of Part 1 crimes in 0.5 mile buffer^d^	141.5 (122.8)	265.7 (244.1)

a Values are mean (standard deviation).

b Within the block group where the residence or park is located.

c Criminal homicide, rape, robbery, aggravated assault, burglary, larceny, motor vehicle theft, arson, and human trafficking.

d Unweighted count of crimes committed within a 0.5 miles street-network buffer of the residence or park.

The relationship between categories of crime rates and crime locations varied with park behavior (Table 3). After accounting for age, sex, race, area income, and the presence of children at home, we found that the number of crimes located around the park was associated with visiting the parks less often (OR = 0.51; 95% CI, 0.34–0.77). Similarly, crimes around the home were also associated with less frequent park visits (OR = 0.56; 95% CI, 0.31–0.82). The number of crimes counted along the route to the park was associated with less frequent park visits to a smaller degree (OR = 0.73; 95% CI, 0.54–1.00) although results were borderline significant. Crime type or crime presence had no significant effect on duration of park visits. Lower park ratings, however, were associated with higher crime presence around the park (OR = 0.33; 95% CI, 0.22–0.50) and more Part I crimes committed around the park (OR = 0.48; 95% CI, 0.33–0.71). Higher crime rates around the home (OR = 0.54; 95% CI, 0.37–0.80) and Part I crimes around the home were also associated with lower park ratings (OR = 0.52; 95% CI, 0.35–0.76). Finally, crime locations were also associated with those who reported they never went to the park. Having high levels of crime around the park was associated with more participants reporting that they never go to the park (OR = 2.81; 95% CI, 1.69–4.68), and the same resulted with higher numbers of Part I crimes committed along the route to the park (OR = 1.54; 95% CI, 1.03–2.32) and around the park (OR = 1.85; 95% CI, 1.17–2.91).

**Table 3:** Effect of Type and Location of Crimes on Park Use, Survey of Respondents (N = 230) on Relationship Between Park Use and Crime, Greensboro, North Carolina, 2013^a^

Variable	Frequency of Park Visits	Duration of Park Visits	Park Rating	Never Goes to Park
**All crimes**				
Home	0.56 (0.38–0.82) [.003]	0.99 (0.68–1.44) [.96]	0.54 (0.37– 0.80) [.002]	1.39 (0.76–1.87) [.44]
Route	0.73 (0.54–1.00) [.05]	1.02 (0.74–1.41) [.89]	0.79 (0.58–1.07) [.13]	1.59 (1.07–2.36) [.02]
Park	0.51 (0.34–0.77) [.001]	0.81 (0.52–1.24) [.33]	0.33 (0.22–0.50) [<.001]	2.81 (1.69–4.68) [<.001]
**Part I crimes^b^**				
Home	0.60 (0.41–0.87) [.01]	0.94 (0.64–1.38) [.76]	0.52 (0.35–0.76) [.001]	1.40 (0.76–1.89) [.45]
Route	0.71 (0.51–0.97) [.03]	0.96 (0.69–1.33) [.79]	0.71 (0.51–0.99) [.045]	1.54 (1.03–2.32)[.04]
Park	0.64 (0.44–0.94) [.02]	0.72 (0.48–1.07)[.10]	0.48 (0.33–0.71) [<.001]	1.85 (1.17–2.91)[.01]

a Values are odds ratio (95% confidence interval) [*P* value].

b Criminal homicide, rape, robbery, aggravated assault, burglary, larceny, motor vehicle theft, arson, and human trafficking.

The distance to the park influenced frequency and duration of park visits, park rating, and the number of participants who reported never going to the park (Table 4). After adjusting for age, sex, race, area income, and the presence of children at home, the only significant associations were distance to the park and duration of park visits, with more distant parks being associated with longer park visits (OR = 1.46; 95% CI, 1.09–1.96). This relationship was unchanged when we controlled for the presence of all crime (OR = 1.46; 95% CI, 1.08–1.96) and the presence of Part I crimes only around the park (OR = 1.46; 95% CI, 1.08–1.96).

**Table 4:** Effect of Distance to Park on Park Use, Survey of Respondents (N = 230) on Relationship Between Park Use and Crime, Greensboro, North Carolina, 2013^a^

Variable	Frequency of Park Visits	Duration of Parks Visits	Park Rating	Never Goes to Park
Without crime	1.07 (0.82–1.42) [.60]	1.46 (1.09–1.96) [.01]	1.01 (0.78–1.29) [.97]	0.82 (0.54–1.24) [.35]
With all crime	1.04 (0.79–1.37) [.78]	1.46 (1.08–1.96) [.01]	0.95 (0.74–1.23) [.70]	0.84 (0.56–1.28) [.42]
Without Part I crime^b^	1.05 (0.80–1.39) [.72]	1.46 (1.08–1.96) [.01]	0.96 (0.74–1.24) [.74]	0.84 (0.55–1.27) [.41]

a Values are odds ratio (95% confidence interval) [*P* value].

b Criminal homicide, rape, robbery, aggravated assault, burglary, larceny, motor vehicle theft, arson, and human trafficking.

## Discussion

Given the important health benefits of park use, park and land use organizations in the United States are advocating for more and better parks within a 10-minute walk from home. However, creating greater access may not lead to greater use, because use also can be affected by the incidence of crime or by perceptions that neighborhoods are unsafe.

Perception of safety is a key element for using parks (27). Violent or egregious crimes are likely to affect safety perception more than minor offenses. As recently investigated by Han et al, (14) violent crimes tend to attract greater media coverage and thus have a greater effect on safety perception. By using the Federal Bureau of Investigation’s Uniform Crime Reporting categories, we were able to focus on violent crimes (Part I offenses) and explore their effects separately. However, the type of crime is likely not the only factor affecting park use. The actual location of the criminal offense is also relevant to the decision making of potential park users. To date, previous studies have not analyzed the exact location of crimes in relation to park use.

By using objective measures of crime and accounting both for the type of crime and the location of the offense, we observed that crimes committed in or around the park were the strongest negative predictor of frequency of park visits, followed closely by the number of crimes around the residence. The number of all crimes located within the shortest route from the residence to the park had no significant effect on park use. The subset of only violent crimes (Part I crimes) located either around the home, around the park, or along the route from home to the park, were all significant predictors of decreased frequency of park use, with violent crimes around the residence having the strongest effects.

Having either more crimes or more violent crimes around the park was most strongly associated with low park ratings by residents. This is important given that previous research found park rating to be a stronger predictor of park use than other factors, such as distance or accessibility (9,28). Our findings suggest that having more crimes in a park might decrease not only how often residents go to the park but also how they view and rate that park, potentially lowering a park’s appeal. Although we found no crime measure that affected duration of park visits, we did find that both violent and nonviolent crimes that occurred around the park and along the route to the park were positively associated with the number of people who said they never went to the park. Finally, our results also suggest that distance to the park affected only the amount of time spent at the park and not the decision whether to go to the park. Interestingly, this association between distance and length of stay appeared to be unchanged when we accounted for crime or violent crimes.

Our sample included older African American women, a group most vulnerable to crime of the populations we studied, and research has shown that the most vulnerable groups tend to take protective action and change behaviors when a threat is perceived (1). Although previous studies showed a strong correlation between crime rates and poverty (13), our sample’s median household income was 28% higher than the Greensboro median. Our finding that proximity to the nearest park was not associated with increased park use is similar to that of previous studies that found no association between park proximity and physical activity in the park (9,17).

Although our study provides some new insights, it has limitations. We used self-reported measures of park use, which could cause recall bias. Our research was cross-sectional in design and had a limited sample, which did not allow for generalization. Additionally, crime data from law enforcement were taken at face value despite known limitations of such data (29). Significant strengths of our study were our use of objective measures of crime and crime types rather than aggregate indexes and our relating the exact location of crimes to 3 dimensions of neighborhood park use that can affect physical activity (park rating, frequency of visits, and duration of visit).

Future research is needed, given our study’s limitations. In particular, researchers are encouraged to explore whether our findings can be duplicated with larger samples in more diverse geographic regions and whether interactions between variables yield more nuanced results between crime, sex, or age and park use. Parks can be an important component of quality of life, but crime can be a barrier. How to reduce crime in and around parks will need to be considered if parks and green spaces are to be a means of promoting physical activity.
